# Human Macrophage Polarization Dynamics on a 3D Fibrous Polydioxanone Mesh In Vitro

**DOI:** 10.3390/jfb17090442

**Published:** 2026-09-02

**Authors:** Ana Laura de Senne Zonta, Biagio Matera, Paulo Tambasco de Oliveira, Isabela Rodrigues Gonsales, Carlos Eduardo Santos Melo, Francesca Rossi, Daniela Bazan Palioto, Benedetta Ghezzi

**Affiliations:** 1 Department of Medicine and Surgery, Center of Dental Medicine, University of Parma, Via Gramsci 14, 43126 Parma, PR, Italy; analaurazonta@usp.br (A.L.d.S.Z.); biagio.matera@unipr.it (B.M.); 2 Department of Oral & Maxillofacial Surgery and Periodontology, Ribeirão Preto School of Dentistry, University of São Paulo—USP, Av. Café s/n, Ribeirão Preto 14040-904, SP, Brazil; isabela.gonsales@usp.br (I.R.G.); cadu-melo@usp.br (C.E.S.M.); 3 Department of Basic and Oral Biology, Ribeirão Preto School of Dentistry, University of São Paulo—USP, Av. Café s/n, Ribeirão Preto 14040-904, SP, Brazil; tambasco@usp.br; 4 Institute of Materials for Electronics and Magnetism, National Research Council (CNR-IMEM), Viale Area delle Scienze 37/A, 43100 Parma, PR, Italy; francesca.rossi@imem.cnr.it

**Keywords:** polydioxanone, macrophage polarization, THP-1-derived macrophages, fibrous biomaterials, biomaterial–immune interactions, periodontal regeneration

## Abstract

The host immune response plays a central role in determining biomaterial performance, particularly through early macrophage activation dynamics at the tissue–biomaterial interface. This study evaluated the response of THP-1-derived human macrophages cultured on a fibrous extracellular matrix-like polydioxanone (PDO) mesh under basal (M0), M1-induced, and M2-induced in vitro conditions. Macrophage responses were assessed by gene expression analysis, immunofluorescence staining of macrophage- and polarization-associated markers, scanning electron microscopy (SEM), and metabolic and viability assays. Distinct stimulus- and time-dependent responses were observed across transcriptional, marker distribution, morphological, and metabolic readouts. Pro-inflammatory gene expression increased under M1-polarizing conditions at 72 h and declined at 96 h, whereas anti-inflammatory and M2-associated markers progressively increased under M2 stimulation, reaching higher levels at 96 h. Immunofluorescence confirmed macrophage differentiation under basal conditions and showed condition-dependent distributions of CD86- and CD206-associated signals, consistent with the transcriptional profiles. SEM revealed stable cell–material interactions and polarization-associated morphological heterogeneity. Metabolic and viability assays confirmed viable cells under all conditions, with activity patterns consistent with induced activation states. Under basal (M0) conditions, macrophages cultured on the PDO mesh maintained a basal phenotype without evidence of marked pro-inflammatory activation. Overall, these findings indicate that fibrous PDO meshes support macrophage viability, differentiation, and stimulus-responsive activation, highlighting their potential as immunologically compatible polymeric platforms for regenerative biomaterial applications.

## 1. Introduction

Biomaterials play a central role in regenerative dentistry, particularly in addressing persistent clinical challenges associated with the reconstruction of bone and periodontal tissues [1]. Although autogenous, allogeneic, and xenogeneic biomaterials remain clinically relevant, their inherent limitations, including donor-site morbidity, restricted availability, and biological variability [2], have driven the development of synthetic alloplastic alternatives [3]. Advances in materials engineering have enabled the fabrication of three-dimensional (3D) constructs with tunable physicochemical and mechanical properties [4], supporting the design of biomaterials that more closely resemble native tissue architecture [5] and provide bioinstructive cues to surrounding cells [6].

The biological performance of implanted biomaterials is largely determined by the host immune response elicited upon implantation [7]. Among the key regulators of this response are macrophages, monocyte-derived and highly plastic myeloid cells that coordinate early inflammatory events following tissue injury [3,8]. These cells integrate microenvironmental cues, including those provided by biomaterials, and translate them into activation programs that influence the balance between inflammation resolution and tissue repair [9]. The extent to which a biomaterial engages macrophage responses is therefore a critical factor influencing integration and regenerative outcomes [10].

Although macrophage activation in vivo is recognized as a dynamic continuum with overlapping phenotypic features, in vitro models commonly distinguish between classically activated (M1) and alternatively activated (M2) phenotypes [11,12]. M1 macrophages are associated with pro-inflammatory activity and expression of mediators such as interleukin-8 (IL-8), interleukin-12 (IL-12), and tumor necrosis factor-alpha (TNF-α) [13]. In contrast, M2 macrophages are linked to anti-inflammatory and pro-regenerative functions, characterized by the expression of interleukin-10 (IL-10), arginase-2 (ARG-2), and the mannose receptor (CD206) [14,15]. These activation states can be experimentally induced using defined cytokine combinations—lipopolysaccharide (LPS) and interferon-γ (IFN-γ) for M1, and interleukin-4 (IL-4) and/or IL-10 for M2—providing a controlled framework for in vitro investigation [16]. Within this context, such models enable systematic assessment of whether biomaterial-derived cues influence macrophage functional trajectories relevant to tissue integration.

During physiological wound healing, macrophages initially adopt a pro-inflammatory activation profile (M1-like) that supports host defense, clearance of debris, and early angiogenic signaling [11]. Progression toward tissue repair requires a timely transition to pro-regenerative states (M2-like) that promote progenitor cell recruitment, extracellular matrix (ECM) deposition, and structural remodeling [17]. A comparable sequence occurs following biomaterial implantation, as the host immune system responds to the implanted material [9]. While an early inflammatory phase is necessary to initiate repair, prolonged M1-associated activity may lead to foreign body reactions characterized by fibrotic encapsulation and impaired integration [11]. The temporal regulation of macrophage activation at the biomaterial interface is therefore a critical factor influencing regenerative outcomes and the progression toward either constructive remodeling or chronic inflammation [9].

Material properties such as surface chemistry, topography, mechanical characteristics, degradation kinetics, and 3D architecture contribute to shaping the local microenvironment that influences cell adhesion, differentiation, and polarization state [10,11]. In particular, biomaterials with fibrous, ECM-like architectures have been associated with more favorable host responses, as their 3D organization and interconnected porosity more closely recapitulate physiological spatial cues [18,19], supporting constructive remodeling and reduced persistence of inflammatory responses [20]. These design considerations are particularly relevant to membrane-based regenerative approaches, in which the biomaterial must simultaneously prevent the ingrowth of non-osteogenic soft tissues, preserve the regenerative space, and provide a biocompatible and bioactive interface that supports cell–material interactions and a favorable early host response [21].

Although collagen membranes remain widely regarded as the clinical reference standard for guided bone regeneration (GBR), their limited mechanical stability and rapid degradation rate may constrain their performance when prolonged barrier function or substantial space maintenance is required [21,22]. Moreover, their natural, frequently xenogeneic origin may introduce source- and processing-related variability, along with a residual risk of immunoreactivity [19]. Such limitations have encouraged the investigation of synthetic bioresorbable polymers, including poly(lactic-co-glycolic acid) (PLGA), polycaprolactone (PCL), and polydioxanone (PDO), whose composition, architecture, mechanical properties, and degradation profiles can be more readily tailored to meet tissue-healing requirements [21].

Among these polymers, PDO warrants particular investigation because it combines flexibility, gradual hydrolytic resorption, low immunogenicity, and established clinical use in absorbable medical devices, providing a strong biomedical rationale for its evaluation as a membrane-based regenerative material [23,24]. Its processing versatility enables the fabrication of fibrous, ECM-like membranes which have demonstrated bone-regenerative performance comparable to collagen membranes in preclinical models [20,25]. PDO-based membranes support stem cell adhesion, migration, proliferation, and osteogenic differentiation in vitro [19] and can be applied as barriers in GBR strategies combined with grafting materials of both natural and synthetic origin in vivo [21,26]. Despite these promising findings, the early macrophage-mediated immune response to fibrous PDO-based membranes, and the extent to which it may contribute to the favorable regenerative outcomes reported, remain insufficiently characterized [27].

Previous work demonstrated that the microarchitecture of electrospun PDO scaffolds, particularly pore size, influences macrophage polarization and macrophage-mediated angiogenesis, with larger pores favoring a more M2-like response; however, this evidence was obtained using primary murine bone marrow-derived macrophages [28]. To our knowledge, no previous in vitro study has systematically characterized the time-dependent response of human macrophages to PDO-based biomaterials intended for GBR. Accordingly, the present study investigated the temporal morphological, immunophenotypic, transcriptional, and metabolic responses of THP-1-derived human macrophages to a fibrous, ECM-like PDO mesh under defined M0, M1, and M2 conditions that recapitulate key aspects of early inflammatory and reparative environments. We hypothesized that the fibrous PDO mesh would support macrophage adhesion and metabolic activity while allowing phenotype- and time-dependent responses to defined polarizing stimuli, including the maintenance or acquisition of M2-associated features, without imposing a sustained pro-inflammatory profile. Although in vitro models do not fully capture the cellular and molecular complexity of the in vivo host response, they enable the controlled interrogation of macrophage–PDO interactions and provide a biologically relevant framework for the rational design of immunomodulatory biomaterials for GBR.

## 2. Materials and Methods

### 2.1. 3D Polydioxanone Mesh

A synthetic, absorbable polymeric mesh composed of polydioxanone (PDO) (Plenum^®^ Guide, Plenum Innovation Center, Jundiaí, São Paulo, Brazil) with dimensions of 30 × 40 × 0.5 mm was used in this study. All specimens were prepared from meshes belonging to the same commercial batch. To perform a morphological characterization of the biomaterial surface, the samples were subjected to scanning electron microscopy (SEM) imaging using a dual-beam Zeiss Auriga Compact system equipped with a GEMINI field-emission SEM column (Carl Zeiss, Oberkochen, Germany) operated at 2 keV. Briefly, SEM micrographs were analyzed using ImageJ Fiji software (version 1.54p; National Institutes of Health, Bethesda, MD, USA) after calibration with the scale bar provided in each image. Fiber diameter was determined by manually measuring the width of 50 randomly selected fibers across representative SEM micrographs using the straight-line tool, and the average fiber diameter was calculated. Mesh porosity was quantified from confocal images acquired using a Sterallis 5 confocal microscope (Leica Mycrosystems, Wetzlar, Germany). Images were analysed in ImageJ by thresholding and image binarization to determine the pore area fraction, which was expressed as the percentage of the total image area occupied by pores.

All specimens were prepared under sterile conditions by cutting the mesh using metal punches, resulting in disc-shaped samples with a diameter of 5 mm. PDO mesh specimens were randomly assigned to three experimental groups before cell seeding:

- **M0:** Unstimulated (no exogenous polarization stimuli);
- **M1:** Pro-inflammatory (M1-like) polarization induced by IFN-γ/LPS stimulation;
- **M2:** Anti-inflammatory/pro-regenerative (M2-like) polarization induced by IL-4 stimulation.

In addition, condition-matched cells-only cultures maintained on tissue culture plastic were included under M0, M1, and M2 conditions as cellular baseline controls. All samples and corresponding cells-only controls were processed in parallel according to group-specific culture conditions and endpoint-specific assay protocols.

### 2.2. THP-1 Human Monocytic Cell Culture and Differentiation

THP-1 human monocytic cells (American Type Culture Collection [ATCC^®^ TIB-202™], Manassas, VA, USA) were expanded in 75 cm^2^ flasks in RPMI-1640 medium (Sigma-Aldrich, St. Louis, MO, USA) supplemented with 10% fetal bovine serum (FBS; Thermo Fisher Scientific, Carlsbad, CA, USA), 1% L-glutamine (Thermo Fisher Scientific, Carlsbad, CA, USA) and 1% penicillin–streptomycin (PenStrep; Sigma-Aldrich, St. Louis, MO, USA), hereafter referred to as complete medium. Cultures were maintained in a humidified incubator at 37 °C and 5% CO_2_, with medium replacement every 48 h during expansion. Cells used in the experiments were derived from the same cell batch and maintained between passages 10 and 15.

**Figure 1:**
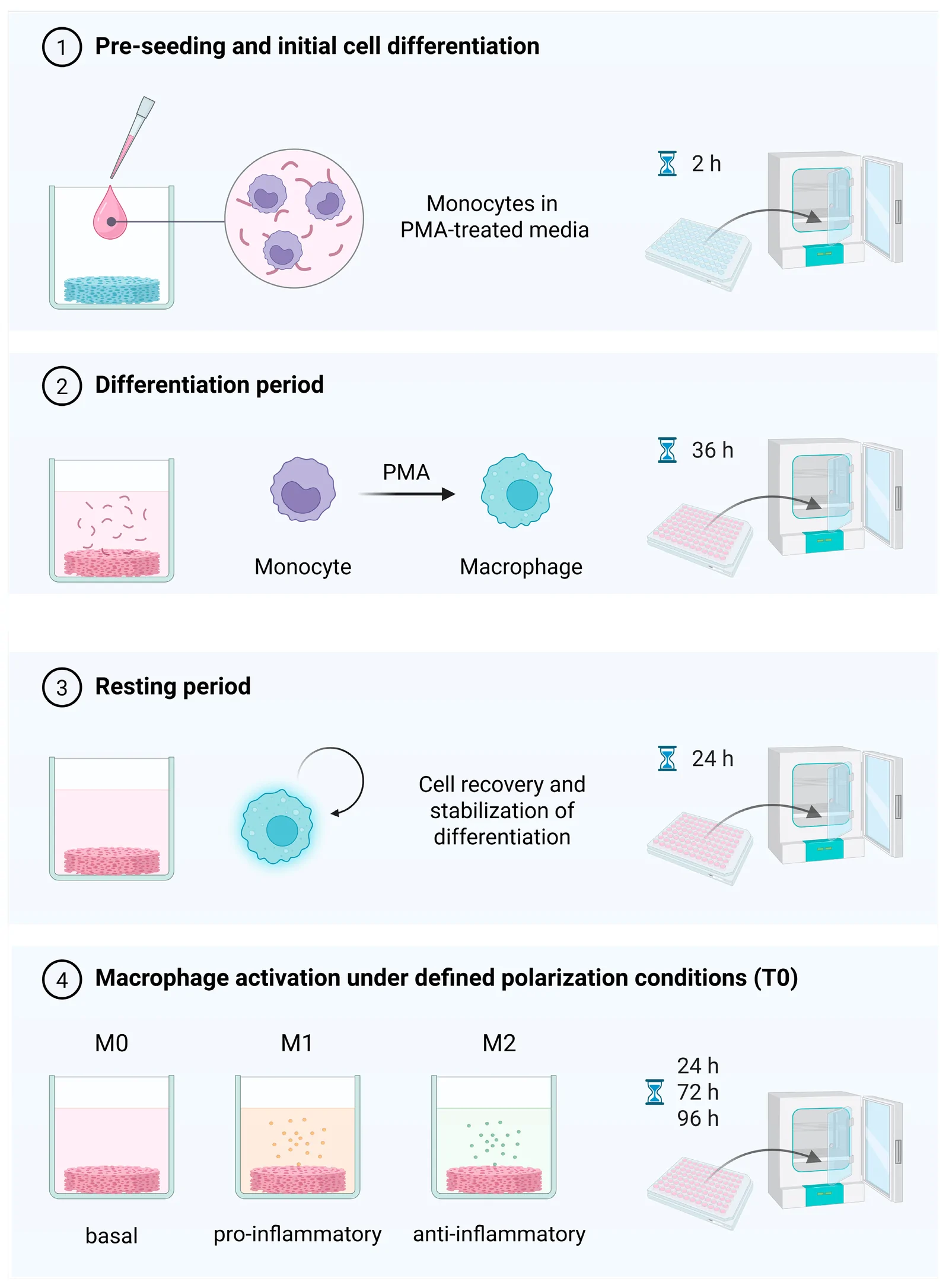
Schematic illustration of THP-1-derived macrophage differentiation and polarization protocol. Created in BioRender. Ghezzi, B. (2026); https://BioRender.com/m3ry4aw.

Cells were resuspended at a density of 6 × 10^6^ cells/mL and immediately prior to seeding, phorbol 12-myristate 13-acetate (PMA; Sigma-Aldrich, St. Louis, MO, USA) was added to the cell suspension (100 ng/mL) to induce initial monocyte-to-macrophage differentiation [16]. Cells were pre-seeded onto each PDO specimen by applying a 30 µL drop of PMA-containing complete medium and incubated for 2 h to promote initial attachment and infiltration into the 3D structure while preventing specimen flotation. Additional PMA-complete medium was then added to each well, and cultures were incubated for a further 36 h. Subsequently, PMA-complete medium was replaced with PMA-free complete medium, and cells were allowed to rest for 24 h to stabilize differentiation and recovery from PMA exposure [16]. The experimental protocol is illustrated in Figure 1.

### 2.3. Macrophage Polarization

Following pre-seeding, differentiation, and resting periods, macrophages in each experimental group were exposed to their respective culture conditions through the addition of fresh complete medium. The M0 group received complete medium without polarization-inducing cytokines, serving as an unstimulated baseline for comparison with cytokine-induced groups.

For cytokine-induced polarization, fresh complete medium was supplemented with the following stimuli (Thermo Fisher Scientific, Carlsbad, CA, USA): IFN-γ and LPS for the M1 group, and IL-4 for the M2 group. All polarization stimuli were used at a final concentration of 100 ng/mL. The polarization stimuli were maintained continuously throughout the experimental period, rather than being applied as a transient stimulation, to sustain the M1 and M2 polarization conditions until the respective experimental endpoints [13].

All groups were otherwise handled identically and underwent the same differentiation protocol, such that cytokine-mediated polarization represented the primary experimental variable. The differentiation and polarization conditions were selected according to previously reported THP-1 macrophage protocols [16] and adapted to the 3D PDO mesh model based on preliminary pilot assays [13].

The time of cytokine addition was defined as time zero for experimental reference, and subsequent time points (24, 72, and 96 h) were established relative to this event. No medium replacement was performed during this period.

### 2.4. Gene Expression Characterization

To assess differential gene expression, total RNA was extracted at 72 and 96 h using the RNeasy^®^ Mini Kit (Qiagen, Hilden, Germany), according to the manufacturer’s instructions. Complementary DNA (cDNA) was synthesized using the High-Capacity cDNA Reverse Transcription Kit (Applied Biosystems; Thermo Fisher Scientific, Vilnius, Lithuania).

Quantitative real-time polymerase chain reaction (qRT-PCR) was performed using TaqMan^®^ assays (Applied Biosystems; Thermo Fisher Scientific, Foster City, CA, USA) and a StepOnePlus™ Real-Time PCR System (Applied Biosystems; Thermo Fisher Scientific, Carlsbad, CA, USA) to quantify the expression of pro-inflammatory genes IL-8 (Hs00174103_m1), TNF-α (cod. Hs00174128_m1), IL-1β (cod. Hs00174097_m1), IL-18 (cod. Hs01030788_m1); the anti-inflammatory gene IL-10 (cod. Hs00961622_m1), and the M2-associated markers CD206 (cod. Hs07288635_g1) and ARG-2 (cod. Hs00982833_m1). Relative gene expression levels were normalized to the housekeeping gene glyceraldehyde-3-phosphate dehydrogenase (GAPDH; cod. Hs02758991_g1). Relative gene expression was calculated using the 2^^−ΔΔCt^ method.

### 2.5. Immunofluorescence Characterization of Polarization-Associated Markers

Immunofluorescence was performed to qualitatively assess the expression of the pan-macrophage marker CD68 and the phenotype-associated markers CD86 and CD206 at 72 and 96 h.

Samples were fixed with 4% paraformaldehyde (PFA; Sigma-Aldrich, St. Louis, MO, USA) for 10 min and washed with phosphate-buffered saline (PBS; Thermo Fisher Scientific, Waltham, MA, USA) before and after a 5 min permeabilization step using 0.1% Triton X-100 (Sigma-Aldrich, St. Louis, MO, USA) at room temperature (RT). Nonspecific binding sites were blocked with PBS containing 10% bovine serum albumin (Sigma-Aldrich, St. Louis, MO, USA) and 3% normal goat serum (Sigma-Aldrich, St. Louis, MO, USA) for 90 min at RT.

Samples were incubated overnight at 4 °C with primary antibodies directed against macrophage phenotype-associated markers. Anti-CD86 antibody [BU63] (mouse monoclonal; 1:150 dilution; ab213044, Abcam, Cambridge, UK) and anti-CD206 (mannose receptor) antibody (rabbit polyclonal; 1:150 dilution; ab64693, Abcam, Cambridge, UK) were used to assess M1- and M2-associated marker expression, respectively. These markers were applied concomitantly to all experimental groups (M0, M1, and M2) to enable qualitative comparison of marker distribution across polarization conditions. In addition, anti-CD68 antibody [KP1] (mouse ascites; 1:150 dilution; NB100-683, Novus Biologicals, Centennial, CO, USA) was used as a macrophage marker in separately stained M0 samples to confirm monocyte-to-macrophage differentiation under basal conditions.

After three PBS washes, appropriate secondary antibodies were applied for 1 h: goat anti-mouse IgG (H+L) Alexa Fluor™ Plus 488 (1:300; A32723, Invitrogen, Thermo Fisher Scientific, Waltham, MA, USA) for detection of mouse-derived primary antibodies (CD68 and CD86), and goat anti-rabbit IgG (H+L) Alexa Fluor™ 555 (1:300; A21428, Invitrogen) for detection of the rabbit-derived primary antibody (CD206). Nuclei were counterstained with 4′,6-diamidino-2-phenylindole (DAPI; 1:1000; FAK100, Merck Millipore, Darmstadt, Germany) for 5 min.

Samples were mounted on glass slides (Thermo Fisher Scientific, Waltham, MA, USA) using Vectashield^®^ anti-fade fluorescence mounting medium (Vector Laboratories, Burlingame, CA, USA) and imaged using a confocal laser scanning microscope (Stellaris 5; Leica Microsystems).

### 2.6. Morphological Characterization

Scanning electron microscopy (SEM) was used for the qualitative assessment of macrophage morphology and cell–material interactions at 96 h. Specimens were rinsed in PBS and fixed with 2.5% glutaraldehyde in 0.1 M sodium cacodylate buffer (Sigma-Aldrich, St. Louis, MO, USA) for 20 min at RT. Samples were then washed in sodium cacodylate buffer for 5 min, dehydrated through graded ethanol solutions (35%, 50%, 70%, 90%, and 100%; 10 min each), and sputter-coated with gold (SCD 040; Balzer Union, Wallruf, Germany). Imaging was performed using a dual-beam Zeiss Auriga Compact system equipped with a GEMINI field-emission SEM column (Carl Zeiss) operated at 2 keV. Subsequently, SEM images were analyzed using ImageJ software after calibration with the scale bar provided in each image. Cellular area, perimeter, and length-to-width ratio were measured across multiple fields of view. In addition, adherent macrophages were manually counted in at least three randomly selected SEM fields per condition using the Cell Counter plugin in ImageJ. Data were expressed as mean ± SD.

### 2.7. Metabolic Activity and Viability Assessment

Metabolic activity and viability were assessed at 24 h and 96 h using the 3-(4,5-dimethylthiazol-2-yl)-2,5-diphenyltetrazolium bromide assay (MTT; Sigma-Aldrich, St. Louis, MO, USA), the CellTiter-Glo^®^ Luminescent Viability Assay (Promega, Madison, WI, USA), and Calcein-AM/Propidium Iodide staining (Thermo Fisher Scientific). Each assay was performed on independent specimens to avoid assay interference and ensure data independence.

For the MTT assay, specimens were incubated for 4 h at 37 °C in fresh medium containing 10 µL of MTT labeling reagent. Subsequently, 100 µL of solubilization solution was added, and plates were incubated overnight under standard culture conditions to ensure complete dissolution of formazan crystals. Absorbance was measured at 570 nm using a Multiskan™ FC microplate reader (Thermo Fisher Scientific, Waltham, MA, USA).

For ATP-based viability assessment, specimens were rinsed in PBS and incubated with a 50:50 mixture of CellTiter-Glo^®^ reagent and RPMI-1640 medium. After shaking for 2 min, lysates were incubated for 10 min at RT in the dark to stabilize luminescence. Luminescence was measured using a GloMax^®^ 20/20 luminometer with dual injectors (Promega, Madison, WI, USA).

For the MTT and CellTiter-Glo^®^ assays, PDO mesh-only specimens without cells were included as assay blanks to account for potential background signals arising from the biomaterial and assay reagents. Raw absorbance or luminescence readings were first corrected by blank subtraction. Blank-corrected values were then normalized to the corresponding polarization- and time-matched tissue culture plastic (TCP) controls and expressed as relative values (test/control ratio), with the corresponding TCP control assigned a value of 1.

Cell viability was further evaluated by Calcein-AM/Propidium Iodide staining to qualitatively assess viable and membrane-compromised cells. At 24 and 96 h, specimens were incubated with 4 µM calcein-AM and 7.5 µM propidium iodide in PBS for 10 min at 37 °C, protected from light. After washing with PBS, samples were fixed in PFA for 20 min to preserve fluorescence prior to imaging. Fluorescence images were acquired using a confocal laser scanning microscope (Stellaris 5; Leica Microsystems, Wetzlar, Germany).

### 2.8. Statistical Analysis

Statistical analyses were performed using GraphPad Prism^®^ 10.0.0 (GraphPad Software, La Jolla, CA, USA). Data are presented as mean ± standard deviation (SD) from three independent experiments. Technical replicates were averaged within each independent experiment, and the independent experiment was considered the statistical unit. Prior to statistical analysis, normality of the residuals from each two-way ANOVA model was assessed using the Shapiro–Wilk test. No statistically significant departure from normality was detected for any of the analysed datasets. Differences between groups were analysed using ordinary two-way analysis of variance (ANOVA), considering polarization condition and time as fixed factors, followed by Tukey’s multiple-comparisons test. Statistical significance was set at *p* < 0.05. For clarity, a complete summary of the two-way ANOVA results has been included as Supplementary Table S1.

## 3. Results

### 3.1. PDO Mesh Exhibits a Fibrous and Porous Morphology Resembling the Extracellular Matrix

The surface topography of the PDO mesh revealed a filamentous structure composed of a non-uniform, interlaced arrangement at the (sub)micrometric scale, closely resembling the fibrous architecture of the ECM.

In this context, the physicochemical and structural properties of the PDO mesh provide an important framework for interpreting the observed biological responses. Previous characterization of the PDO mesh has demonstrated a semicrystalline structure (crystallinity = 52.86 ± 2.97%) with adequate thermal stability (Tm = 107.35 °C), pronounced swelling capacity (≈425% thickness increase within 24 h), and favorable mechanical properties, including an elastic modulus of 30.1 ± 6.25 MPa, tensile strength of 3.92 ± 0.28 MPa, and elongation at break of 287.9 ± 34.68% [19]. Morphometric analysis of confocal and SEM images showed an average fiber diameter of 2.17 ± 0.62 µm and membrane porosity of 73.76 ± 2.86%.

### 3.2. Polarization Conditions Drive Distinct Pro- and Anti-Inflammatory Gene Expression Profiles over Time

**Figure 2:**
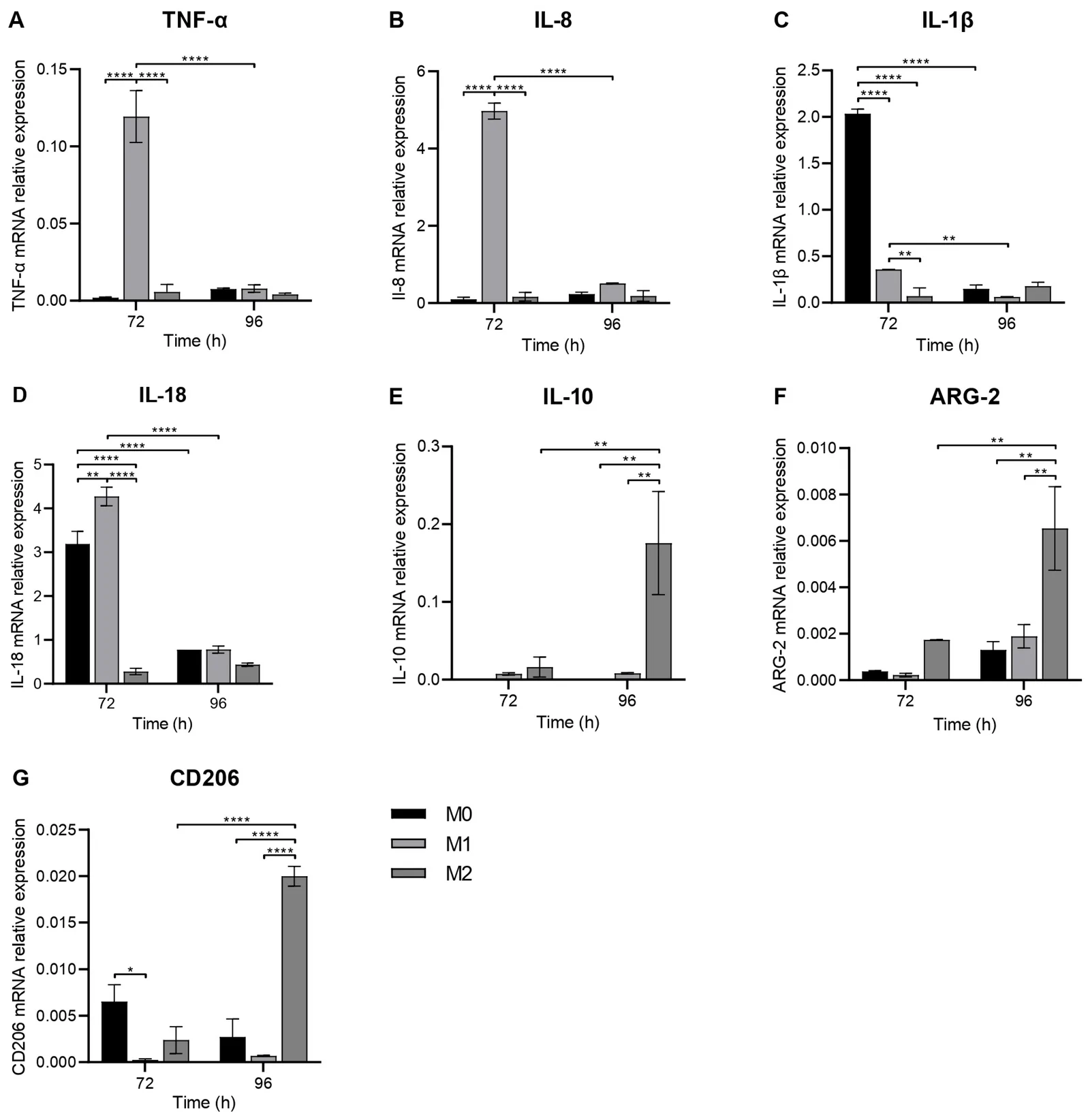
Relative mRNA expression of macrophage polarization-associated markers under M0, M1, and M2 conditions: the pro-inflammatory markers TNF-α (**A**), IL-8 (**B**), IL-1β (**C**), and IL-18 (**D**); the anti-inflammatory marker IL-10 (**E**); and the M2-associated markers ARG-2 (**F**) and CD206 (**G**) was assessed by qRT-PCR at 72 and 96 h. TNF-α and IL-8 showed their highest expression in M1 at 72 h, whereas IL-1β and IL-18 exhibited distinct polarization-dependent profiles but similarly decreased over time in M0 and M1; in contrast, IL-10, ARG-2, and CD206 increased under M2 conditions, with higher expression at 96 h. Relative gene expression was normalized to GAPDH and calculated relative to the corresponding M0 control using the 2^^−ΔΔCt^ method. Statistical analysis was performed using two-way ANOVA followed by Tukey’s multiple-comparisons test. Data are presented as the mean ± SD. Exact *p*-values for significant comparisons not represented by **** *p* < 0.0001 were as follows: Panel (**C**): ** *p* = 0.0068 (M1 72 h vs. M1 96 h), ** *p* = 0.0077 (M1 72 h vs. M2 72 h); Panel (**D**): ** *p* = 0.0032 (M0 72 h vs. M1 72 h); Panel (**E**): ** *p* = 0.0089 (M2 72 h vs. M2 96 h), ** *p* = 0.0055 (M0 96 h vs. M2 96 h), ** *p* = 0.0069 (M1 96 h vs. M2 96 h); Panel (**F**): ** *p* = 0.0063 (M2 72 h vs. M2 96 h), ** *p* = 0.004 (M0 96 h vs. M2 96 h), ** *p* = 0.0074 (M1 96 h vs. M2 96 h); Panel (**G**): * *p* = 0.0213 (M0 72 h vs. M1 72 h).

Gene expression analysis revealed time- and polarization-dependent differences in macrophage-associated gene expression across experimental conditions (Figure 2).

The pro-inflammatory markers TNF-α and IL-8 showed their highest expression under M1-polarizing conditions at 72 h, followed by a significant decrease at 96 h (*p* < 0.0001), while expression remained low in M0 and M2 at both time points, with no significant temporal changes in either group. At 72 h, both genes showed significantly higher expression in M1 than in M0 and M2 (*p* < 0.0001). By 96 h, no significant differences were detected among M0, M1, and M2 groups (Figure 2A,B).

The pro-inflammatory markers IL-1β and IL-18 also exhibited time-dependent changes, while displaying distinct expression profiles across polarization conditions (Figure 2C,D). From 72 to 96 h, expression of both genes decreased significantly in M0 (*p* < 0.0001 for both) and M1 (IL-1β: *p* = 0.0068; IL-18: *p* < 0.0001), with no significant temporal changes observed in M2. At 72 h, all pairwise comparisons among M0, M1, and M2 were significant for both genes; however, IL-1β expression was highest in M0 (M0 vs. M1 and M2: *p* < 0.0001; M1 vs. M2: *p* = 0.0077), whereas IL-18 expression was highest in M1 (M0 vs. M1: *p* = 0.0032; M0 vs. M2 and M1 vs. M2: *p* < 0.0001). By 96 h, no significant differences were detected among groups for either gene.

In contrast to the temporal decreases observed for the pro-inflammatory markers, the anti-inflammatory (IL-10) and M2-associated (ARG-2 and CD206) markers displayed significant increases from 72 to 96 h under M2-polarizing conditions (IL-10: *p* = 0.0089; ARG-2: *p* = 0.0063; CD206: *p* < 0.0001), while no significant temporal changes were observed in M0 or M1 (Figure 2E–G). At 72 h, no significant intergroup differences were detected for IL-10 or ARG-2, whereas CD206 expression was significantly higher in M0 than in M1 (*p* = 0.0213), with no other significant pairwise differences. At 96 h, all three markers showed significantly higher expression in M2 than in M0 (IL-10: *p* = 0.0055; ARG-2: *p* = 0.004; CD206: *p* < 0.0001) and M1 (IL-10: *p* = 0.0069; ARG-2: *p* = 0.0074; CD206: *p* < 0.0001). No significant differences were detected between M0 and M1.

### 3.3. Polarization-Associated Marker Distribution Differs Across Conditions in Macrophages on PDO Meshes

**Figure 3:**
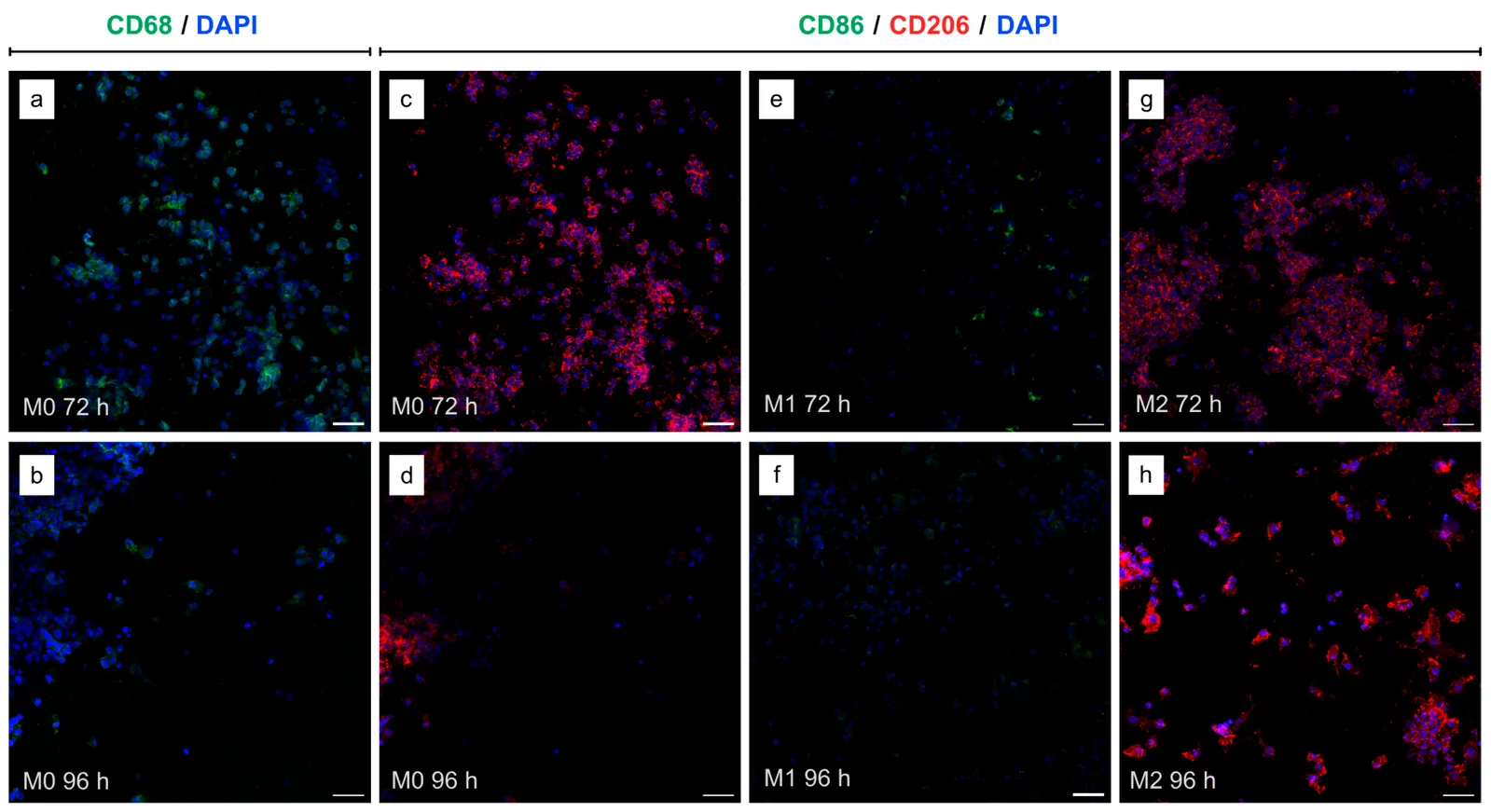
Representative immunofluorescence images of macrophages cultured on PDO meshes at 72 h and 96 h under M0, M1, and M2 conditions. CD68 (green; pan-macrophage marker) is shown in M0 samples (**a**,**b**). In the remaining panels, CD86 (green; M1-associated marker) and CD206 (red; M2-associated marker) were applied concomitantly to all polarization conditions (**c**–**h**). Nuclei were counterstained with DAPI (blue). Magnification: 40×. Scale bar: 50 μm.

Distinct distributions of macrophage and polarization-associated markers were observed across conditions and time points (Figure 3). DAPI-stained nuclei were consistently detected under all conditions, with preserved nuclear morphology. In M0 samples, CD68-associated fluorescence confirmed the presence of differentiated macrophages distributed across the mesh surface (Figure 3a,b). A less extensive CD68 signal was observed at 96 h compared with 72 h, likely reflecting a physiological reduction in cell number over time rather than a decrease in CD68 expression. As THP-1-derived macrophages are terminally differentiated cells that do not proliferate, the lower signal at 96 h is consistent with the reduced metabolic activity observed at this time point and may result from gradual cell loss due to physiological cell death and/or partial cell detachment from the PDO mesh during prolonged culture.

At 72 h, the M0 group exhibited a broad cellular distribution with a predominance of CD206-associated fluorescence relative to CD86 (Figure 3c). The M1 group appeared less densely populated and displayed weak, sparsely distributed CD86-associated signal (Figure 3e). In contrast, the M2 group showed an extensive fluorescence pattern, with CD206-associated signal distributed across a densely populated field, without detectable CD86-positive cells (Figure 3g).

At 96 h, condition-specific changes in polarization-associated fluorescence were observed relative to 72 h (Figure 3d,f,h). In M0, CD206-associated fluorescence was less extensive than at the earlier time point (Figure 3d). In the M1 group, CD86 signal remained limited and appeared less evident at 96 h (Figure 3f). M2 samples showed a more dispersed cellular distribution and fewer densely populated clusters than at 72 h, while CD206-associated fluorescence remained prominent and appeared visually more intense in the representative 96 h field (Figure 3h).

Cells exhibiting DAPI-stained nuclei in the absence of detectable CD86 or CD206 signals were observed in the M1 group (Figure 3e,f) and, less frequently, in M0 samples (Figure 3d). Overall, CD206-associated fluorescence predominated over CD86 under M0 and M2 conditions, whereas M1 samples displayed limited CD86-associated staining at both time points.

### 3.4. THP-1-Derived Macrophage Morphology Varies with Polarization on PDO Meshes

**Figure 4:**
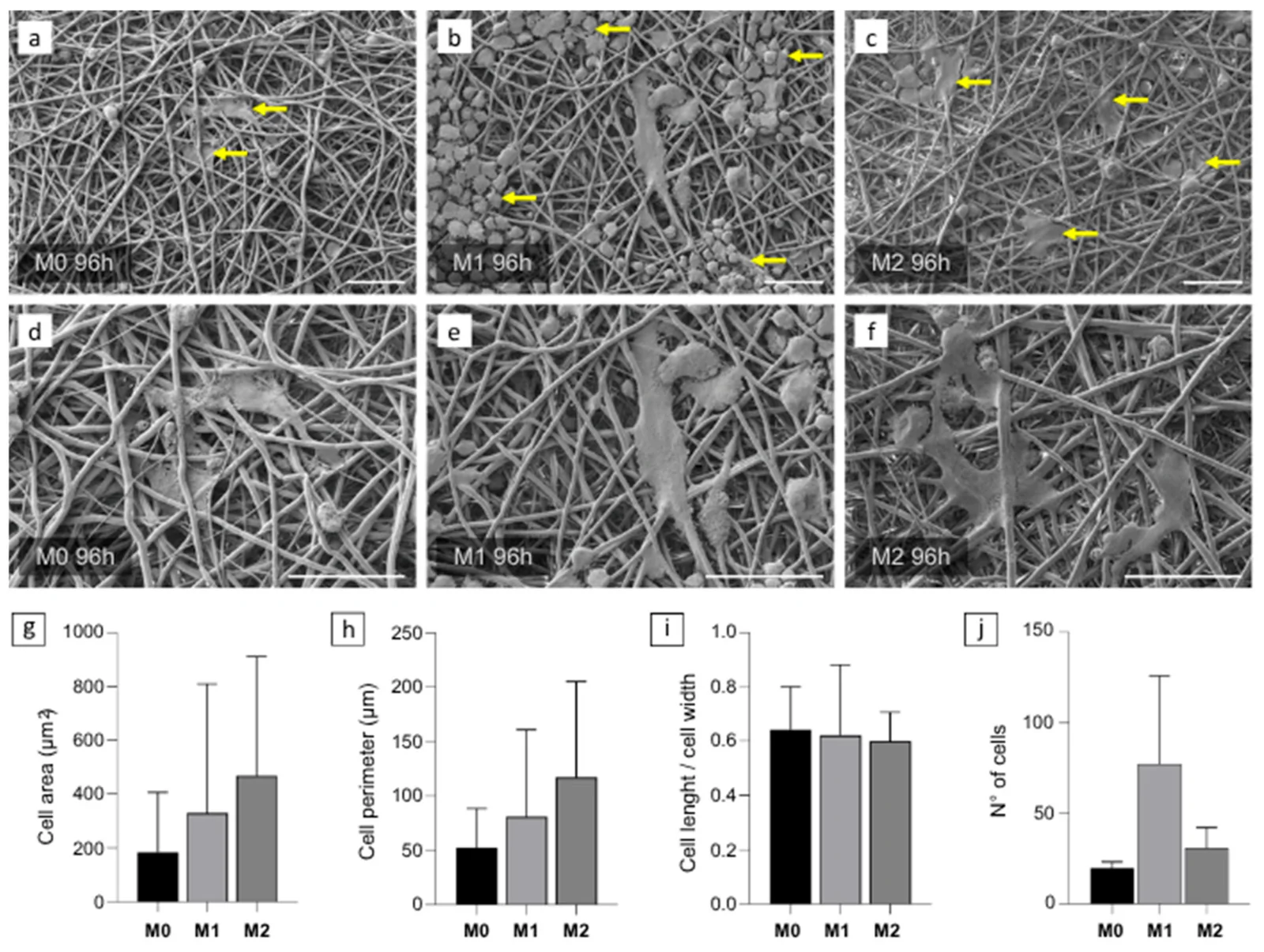
Representative SEM micrographs and quantitative analysis of adherent macrophages cultured on PDO meshes for 96 h under M0-, M1-, and M2-polarizing conditions. Low-magnification views ((**a**–**c**); 1000×) and higher-magnification views ((**d**–**f**); 2000×) illustrate cell adhesion to the fibrous network and polarization-associated morphological patterns. Yellow arrows highlight representative macrophages displaying the characteristic morphology observed under each polarization condition. M0 samples show sparsely distributed cells with limited spreading (**a**,**d**), M1 samples exhibit predominantly rounded cells forming clustered regions (**b**,**e**), and M2 samples display more spread and stellate-like morphologies, with cellular extensions bridging adjacent filaments (**c**,**f**). Scale bar: 40 μm. Quantitative morphometric analysis included (**g**) cell area, (**h**) cell perimeter, (**i**) cell length-to-width ratio, and (**j**) average number of adherent macrophages per SEM field under M0, M1, and M2 conditions. These parameters were quantified from SEM images using ImageJ software. Data are presented as mean ± SD. No statistically significant differences were observed among M0, M1, and M2 groups for any of the measured morphometric parameters.

SEM micrographs demonstrated that macrophages adhered to the PDO mesh and established close contact with its fibrous, interlaced filament network after 96 h of initial polarization induction (Figure 4). The mesh displayed a non-uniform, porous architecture at the (sub)micrometric scale, resembling an ECM-like fibrous structure, within which cells were anchored to individual filaments and extended across adjacent fibers, indicating stable physical interaction with the substrate.

Across all polarization conditions, macrophages displayed both rounded or ovoid and elongated or spread morphologies on the same substrate, with differences in their distribution among groups. In M0 samples, cells appeared more sparsely distributed (Figure 4a), with isolated adherent macrophages exhibiting limited spreading (Figure 4d). In M1 samples, rounded/ovoid morphologies were more prevalent and frequently appeared in closely juxtaposed regions, forming clustered areas within the mesh (Figure 4b). In contrast, M2 samples more commonly displayed spread and stellate-like cells with extended processes bridging multiple filaments (Figure 4c,f). To complement the qualitative assessment, quantitative morphometric analysis was performed by measuring cell area, perimeter, and length-to-width ratio across multiple SEM fields of view (Figure 4g–i). Although mean values showed some variation among polarization conditions, no statistically significant differences were observed for cell area, cell perimeter, or cell length-to-width ratio among M0, M1, and M2 groups. These findings indicate that the polarization-associated morphological patterns observed by SEM were not accompanied by significant differences in the measured quantitative morphometric parameters. Similarly, quantitative analysis of the number of adherent macrophages using the Cell Counter plugin in ImageJ showed no statistically significant differences among the three groups (Figure 4j).

### 3.5. Macrophage Metabolic Activity and Viability Vary with Polarization Conditions

**Figure 5:**
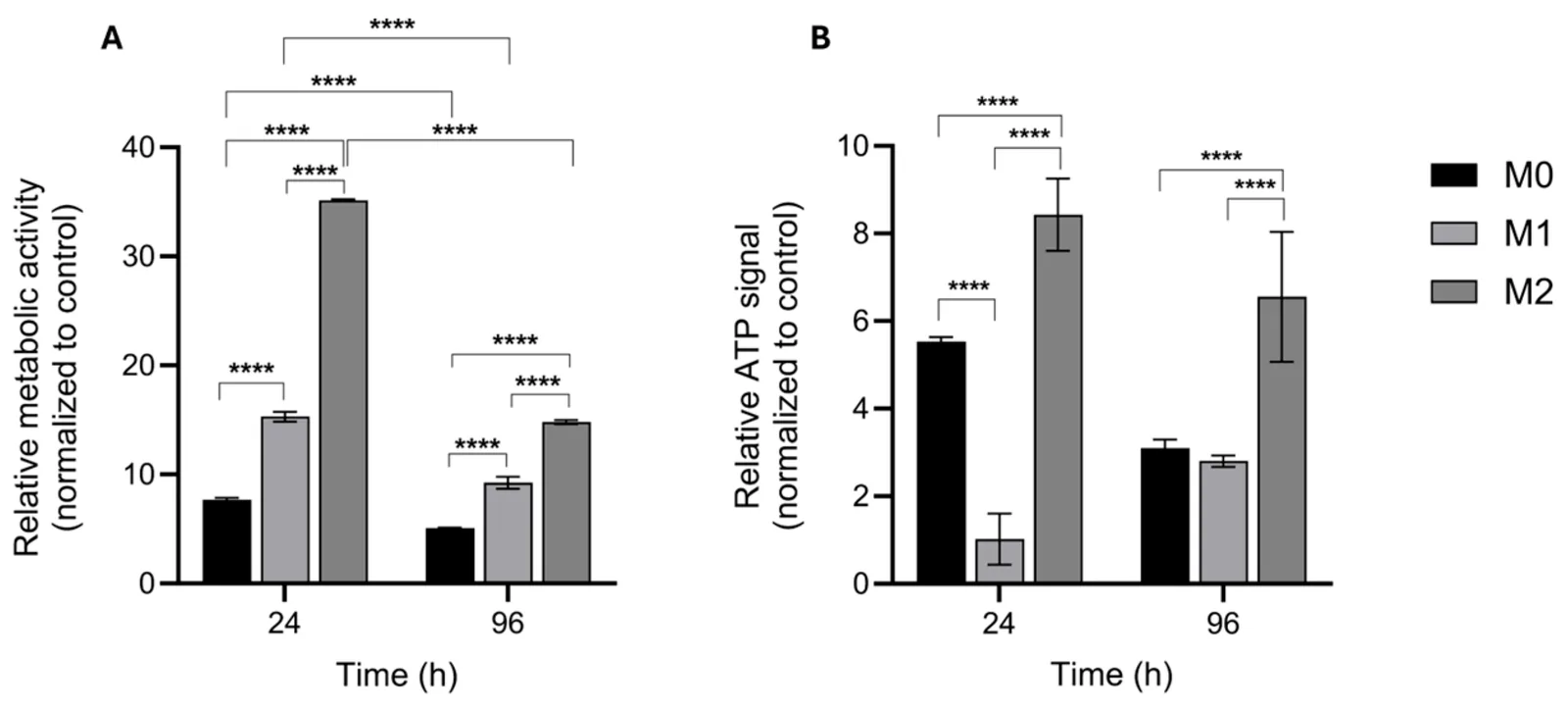
Relative metabolic activity and ATP-based viability of macrophages cultured on PDO meshes under M0, M1, and M2 polarization conditions at 24 h and 96 h. (**A**) MTT assay showing significant intergroup differences at both time points and reduced relative metabolic activity at 96 h compared with 24 h across all groups. (**B**) CellTiter-Glo^®^ assay showing significant intergroup differences, with stable ATP levels over time. Following blank subtraction, assay values obtained from cells cultured on PDO meshes were normalized to the corresponding polarization- and time-matched tissue culture plastic (TCP) controls and expressed as test-to-control ratios. Data are presented as mean ± SD. Panel (**A**): **** *p* < 0.0001; Panel (**B**): **** *p* < 0.0001.

Metabolic activity and viability assays confirmed the presence of viable macrophages across all experimental conditions (Figure 5), without evidence of cytotoxic effects under basal or cytokine-induced stimulation. Distinct activity patterns were observed across polarization conditions. In both assays, macrophages under M2-polarizing conditions exhibited higher activity than M0 and M1 at both time points. Differences between M0 and M1 were assay-dependent. In the MTT assay (Figure 5A), M1 showed higher metabolic activity than M0 at both time points, whereas in the CellTiter-Glo^®^ assay (Figure 5B), M0 displayed higher ATP levels than M1 at 24 h. Over time, metabolic activity decreased from 24 to 96 h in all groups (Figure 5A), while ATP levels remained stable (Figure 5B), consistent with preserved cell viability.

**Figure 6:**
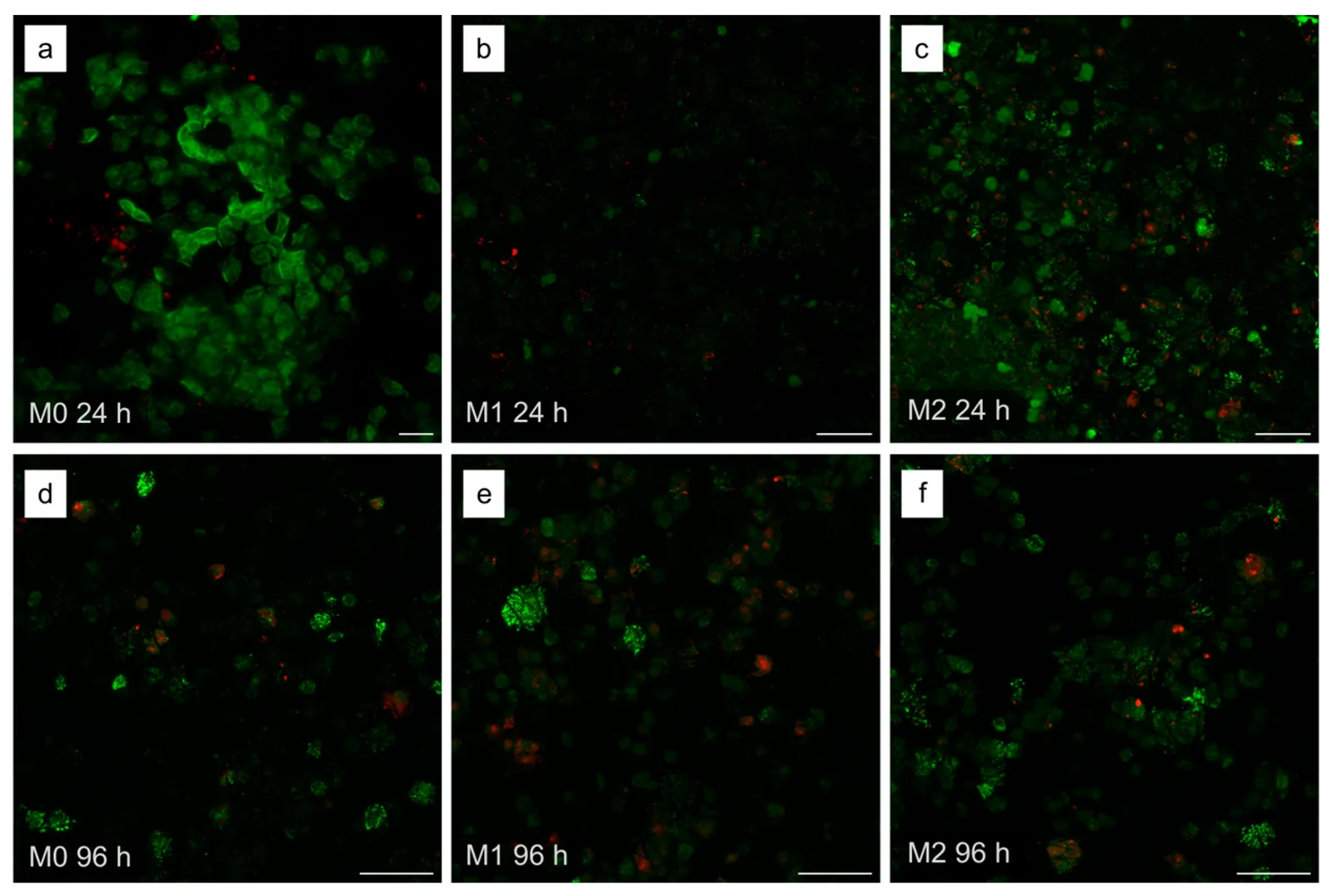
Representative Calcein-AM/Propidium Iodide fluorescence images illustrating viable and dead macrophages cultured on PDO meshes at 24 (**a**–**c**) and 96 h (**d**–**f**) under M0 (**a**,**d**), M1 (**b**,**e**), and M2 (**c**,**f**) conditions. Green fluorescence indicates viable cells distributed throughout the fibrous mesh architecture, while red fluorescence indicates dead or membrane-compromised cells. Scale bar: 50 μm.

Live/dead staining further confirmed the presence of viable cells within the PDO mesh under all conditions (Figure 6). At 24 h, M0 showed prominent Calcein-associated signal concentrated in large, compact clusters (Figure 6a), whereas M2 displayed a broader distribution across the field (Figure 6c); M1 showed sparse and limited signal (Figure 6b). At 96 h, viable cells remained detectable in all groups, with distinct temporal patterns (Figure 6d–f). In both M0 and M2, Calcein-associated fluorescence was less extensive and distributed in more discrete regions than at 24 h (Figure 6d,f), whereas M1 retained a localized pattern, with fluorescent clusters becoming more discernible at the later time point (Figure 6e). Scattered PI-positive cells were also observed across conditions at both time points.

## 4. Discussion

Macrophages are central regulators of the host response to implanted biomaterials [7], coordinating early inflammatory events [8] while influencing the subsequent progression toward either constructive tissue remodeling or persistent inflammation [9]. Within this framework, the present study investigated how THP-1-derived human macrophages respond to a clinically relevant fibrous PDO mesh under defined basal, pro-inflammatory, and anti-inflammatory in vitro conditions over time. Across transcriptional, immunophenotypic, morphological, and metabolic levels, the results consistently indicate that the PDO mesh supports macrophage viability and adhesion while preserving stimulus-responsive polarization dynamics.

In this context, the physicochemical and structural properties of the PDO mesh provide an important framework for the interpretation of the observed biological responses. Previous characterization of the PDO mesh has demonstrated a semicrystalline structure with adequate thermal stability, pronounced swelling capacity, and favorable mechanical properties [19]. In addition, its fibrous architecture preserves structural integrity upon hydration and can be readily combined with particulate grafts [29] and 3D-printed scaffolds [20,26,30], supporting its application as a resorbable barrier in GBR strategies. Although these properties have been linked to favorable biological performance in experimental and clinical settings [19,20,25,29,30,31,32,33,34], prior studies have primarily focused on general biocompatibility, degradation kinetics, or stem cell interactions [19,20,26], whereas macrophage responses to PDO meshes remain comparatively underexplored [28]. When immune-related outcomes are reported, they are often derived from animal models or based on nonspecific inflammatory readouts, thereby limiting mechanistic insight into how this clinically relevant polymer interacts with human immune cells that regulate inflammatory and reparative processes [27,28]. To the best of our knowledge, this study provides the first systematic evaluation of human macrophage polarization dynamics in response to PDO meshes in vitro.

In the present study, macrophages cultured on PDO showed distinct stimulus- and time-dependent responses to the imposed polarization conditions at the transcriptional level. The pro-inflammatory genes TNF-α and IL-8 showed their highest expression at 72 h under M1 conditions, followed by a marked decline at 96 h. The elevated TNF-α and IL-8 expression observed at 72 h is consistent with previous findings in PMA-differentiated THP-1 macrophages subjected to a 24 h resting period followed by 72 h polarization with IFN-γ/LPS, which significantly increased the expression of both genes relative to unstimulated M0 cells [34]. The interpretation of IL-8 as a pro-inflammatory readout, rather than a phenotype-specific M1 marker, is further supported by studies in THP-1 cells showing enhanced IL-8 mRNA expression following IFN-γ/LPS stimulation [35]. Notably, in the present study, the decline in TNF-α and IL-8 occurred despite the continuous presence of IFN-γ/LPS throughout the experimental period and therefore cannot be attributed to withdrawal of the polarizing stimuli. Rather, this temporal profile likely reflects the intrinsic regulation of macrophage activation under sustained pro-inflammatory stimulation. Adaptive regulatory mechanisms, including negative feedback pathways and endotoxin tolerance, have been described in macrophages exposed to prolonged pro-inflammatory stimulation and are associated with attenuation of inflammatory gene expression [16,36].

The pro-inflammatory panel further revealed marker- and time-dependent regulation rather than a uniform M1-associated response. At 72 h, IL-1β was significantly enriched in M0 relative to both polarized conditions, whereas IL-18 was highest in M1 and was also significantly higher in M0 than in M2. This dissociation is consistent with observations in PMA-differentiated THP-1 cells showing distinct IL-1β and IL-18 transcriptional responses despite their shared involvement in inflammasome-dependent cytokine maturation [37]. Accordingly, the lack of preferential IL-1β expression in M1 does not contradict the broader pro-inflammatory transcriptional profile observed at 72 h. This pattern also contrasts with the greater persistence of IL-1β relative to TNF transcription reported following LPS stimulation in THP-1 cells [38], further underscoring the context- and marker-dependent kinetics of inflammatory gene expression. Conversely, the concomitant expression of IL-1β and IL-18 in M0 should not, in isolation, be interpreted as a coordinated M1-associated response or as evidence of a pro-inflammatory effect of the PDO mesh. In a methodologically close THP-1 model, Bianconi et al. (2024) showed that electrically stimulated M0 macrophages upregulated IL-1β and IL-8 concurrently with M2-associated genes [39], consistent with the mixed transcriptional profile observed in the present study.

In contrast, the anti-inflammatory/M2-associated transcriptional response developed progressively under IL-4 stimulation. At 72 h, the M2 condition was not yet characterized by preferential expression of IL-10, ARG-2, or CD206; however, all three genes increased significantly from 72 to 96 h and, at 96 h, were significantly higher in M2 than in both M0 and M1. This delayed transcriptional pattern contrasts with the earlier and transient pro-inflammatory response observed under M1 conditions, revealing distinct temporal kinetics of macrophage polarization on the PDO mesh. IL-10 and CD206 are established readouts of anti-inflammatory and alternatively activated macrophage programs and are commonly used to characterize IL-4-responsive phenotypes in THP-1-derived macrophages [16]. Importantly, temporal dependence of M2-associated markers has also been demonstrated in primary human macrophages, in which IL-4-induced CD206 expression became progressively more evident over time and IL-10 production emerged at later stages of polarization [40]. The parallel increase in ARG-2 adds an immunometabolic dimension to this response. Although ARG-2 is not considered a canonical human M2 marker, it has emerged as a regulator of anti-inflammatory macrophage reprogramming. Dowling et al. (2021) identified ARG-2 as an IL-10-regulated mitochondrial protein that supports oxidative metabolism and contributes to the attenuation of inflammatory signaling, including in THP-1 macrophages [41].

Although THP-1-derived macrophages represent one of the most widely adopted reproducible in vitro models for investigating macrophage polarization and biomaterial–immune interactions, they do not fully recapitulate the biological complexity of primary human monocyte-derived macrophages (MDMs). Differences in donor-specific variability, activation kinetics, cytokine secretion, phenotypic marker expression, and responsiveness to polarizing stimuli have been reported between the two models [16]. Direct comparisons further indicate that polarization-associated transcriptional responses in THP-1-derived macrophages may differ in magnitude and, for some markers, in their relationship with protein secretion compared with primary MDMs [42]. Importantly, these differences are relevant to the temporal pro-inflammatory profile observed here. In primary human MDMs stimulated with LPS/IFN-γ, TNF transcription reaches its highest levels at early time points and subsequently declines, whereas IL-1β expression remains elevated over a broader temporal window [40]. In contrast, in the present THP-1/PDO model, TNF-α remained strongly enriched under M1 conditions at 72 h, while IL-1β was not preferentially expressed in M1 at this time point. Therefore, although the temporal expression patterns observed in the present study provide insight into macrophage responses to the PDO mesh under controlled experimental conditions, their interpretation should account for the intrinsic limitations of the THP-1 model. Validation in primary human macrophages would further strengthen the biological and translational relevance of these findings.

Immunofluorescence extended the transcriptional characterization by revealing the spatial distribution of macrophage- and polarization-associated markers across the PDO mesh. In M0 samples, CD68-associated fluorescence confirmed the presence of differentiated macrophages distributed across the mesh surface under basal conditions, indicating successful monocyte-to-macrophage differentiation on the substrate. By 96 h, both CD68 and CD206 signals were less extensive than at 72 h, while CD206 remained more prominent than CD86 at both time points. Rather than recapitulating either experimentally induced profile, M0 combined the expression of selected pro-inflammatory genes (IL-1β and IL-18) with evidence of CD206 at both transcriptional and immunofluorescence levels, while lacking both the coordinated TNF-α/IL-8 response observed under M1 conditions and the progressive IL-10/ARG-2/CD206 response induced by IL-4. This pattern is consistent with the recognized plasticity of macrophages at biomaterial interfaces, where activation-associated features may overlap without defining a discrete M1 or M2 state [17]. In THP-1 macrophages, Rynikova et al. (2023) further showed that expression of selected M2-associated genes, including MRC1, did not necessarily coincide with establishment of a coordinated M2 transcriptomic program [43], supporting the interpretation of CD206 in M0 as one component of a broader mixed profile rather than evidence of M2 polarization.

Under M1 conditions, CD86-associated fluorescence was weak and sparsely distributed at 72 h and appeared less discernible at 96 h, whereas the broader pro-inflammatory transcriptional program was clearly evident at 72 h. The presence of DAPI-positive nuclei without detectable CD86 or CD206, particularly in M1 and to a lesser extent in M0, further indicates that these two polarization-associated markers did not encompass the full phenotypic spectrum of the macrophage populations. This pattern is compatible with the marker- and time-dependent nature of macrophage polarization: CD86 surface expression varies markedly over time during LPS/IFN-γ polarization in primary human macrophages, while time-course analyses in THP-1 macrophages demonstrate early induction followed by progressive attenuation of several M1-associated transcripts [40,44]. In contrast, M2 samples showed a more dispersed cellular distribution at 96 h, while CD206-associated fluorescence remained prominent at both time points and appeared visually more intense in the representative 96 h field. These qualitative CD206 observations were noted alongside the later coordinated increase in IL-10, ARG-2, and CD206 transcripts, supporting progressive development of the IL-4-responsive phenotype. This delayed coordination of the M2-associated program is consistent with previous THP-1 studies showing that MRC1/CD206 and other IL-4-responsive markers become more evident after longer cytokine exposure, while their magnitude is also influenced by the preceding PMA differentiation and rest conditions [43,45]. Nevertheless, because immunofluorescence was assessed qualitatively, these patterns should be interpreted as phenotypic and spatial context for the transcriptional findings rather than as quantitative measures of polarization-marker expression.

Beyond phenotype-associated marker distribution, SEM provided direct evidence of macrophage interaction with the fibrous PDO architecture at 96 h. Cells from all polarization conditions remained adherent to the interlaced network, establishing close contact with individual filaments and frequently spanning adjacent fibers. This pattern indicates well-established cell–material interactions at 96 h and supports the ability of the fibrous architecture to accommodate macrophage attachment [13]. Although representative fields suggested condition-associated morphological tendencies, with more limited spreading in M0, a greater prevalence of rounded or ovoid configurations in M1, and more frequent spread or stellate-like cells with cytoplasmic extensions in M2, these visual patterns were not supported by significant differences in cell area, perimeter, length-to-width ratio, or number of adherent cells. Such qualitative tendencies remain consistent with prior evidence associating the morphology and distribution of THP-1-derived macrophages with their activation states [28,46], while also underscoring that cell shape is not a phenotype-specific readout in THP-1-derived macrophages and may additionally be influenced by local interactions with the fibrous substrate [13].

Complementing the morphological evidence of macrophage attachment, metabolic and viability assays supported the maintenance of metabolically active cells across all experimental conditions, consistent with previous evidence of PDO biocompatibility [19,24]. M2-polarizing conditions were associated with higher metabolic activity in both assays, whereas differences between M0 and M1 varied depending on the endpoint measured. The divergent trends observed between the MTT and CellTiter-Glo^®^ assays should be interpreted in light of the distinct biological parameters measured by the two assays, which should not be considered interchangeable indicators of cell viability. The MTT assay primarily reflects the activity of NAD(P)H-dependent oxidoreductases, predominantly mitochondrial dehydrogenases, and is therefore sensitive to changes in cellular metabolic activity. In contrast, the CellTiter-Glo^®^ assay quantifies intracellular ATP content, providing a readout more directly related to the cellular energy state and the number of metabolically active viable cells. During classical M1 macrophage activation, profound metabolic reprogramming occurs, characterized by increased glycolytic flux and altered mitochondrial function [47]. Although oxidative phosphorylation is reduced, activated M1 macrophages can retain elevated oxidoreductase activity and increased production of reducing equivalents, which may enhance tetrazolium reduction and consequently increase the MTT signal without necessarily indicating increased ATP production or a greater number of viable cells. Thus, the higher MTT activity observed in M1 macrophages at 24 h likely reflects their activated metabolic phenotype rather than a greater number of viable cells. Conversely, the higher ATP levels detected in M0 macrophages by the CellTiter-Glo^®^ assay at 24 h are consistent with their basal metabolic state and preserved ATP generation under unstimulated conditions. Taken together, these findings indicate that the apparent discrepancy between the two assays reflects activation of state-specific metabolic programs rather than a contradiction in cell viability. The decline in MTT signal over time, together with stable ATP levels, further suggests that reduced metabolic activity at later time points reflects metabolic adaptation rather than loss of viability. Calcein-AM/Propidium Iodide staining provided complementary spatial evidence, showing persistent viable cells across all conditions despite condition-specific changes in their distribution over time. Although the spatial patterns differed more clearly among conditions at 24 h, they appeared less divergent by 96 h, while viable cells remained evident in all groups. Together, these findings support the interpretation that the temporal changes in metabolic readouts primarily reflect adaptation of macrophage metabolic activity rather than cytotoxicity.

Viewed from a regenerative biomaterials perspective, these findings support macrophage compatibility rather than active immunomodulation by the PDO mesh. Notably, the experimental system did not sustain a uniformly elevated pro-inflammatory transcriptional response over time, even under continuous IFN-γ/LPS stimulation. Although the absence of persistent inflammatory activation is relevant to biomaterial integration, it should not be equated with active pro-resolving immunomodulation or attributed specifically to the PDO mesh. Actively immunomodulatory biomaterials instead seek to exploit material-derived physical or biochemical cues to direct macrophage functions toward inflammation resolution and constructive tissue remodeling [8,11]. Previous evidence that fiber and pore architecture can modulate macrophage responses to electrospun PDO further suggests that structural optimization may provide a route toward more active immunomodulatory control [28]. However, because only one PDO architecture was evaluated here, whether structural optimization can actively direct macrophage function cannot be established from the present study.

## 5. Conclusions

The present in vitro model provides a controlled framework for the systematic investigation of early macrophage–material interactions and reproducible induction of defined activation states. In this context, the present PDO mesh should be regarded as a macrophage-compatible substrate that preserves responsiveness to exogenous polarizing cues, rather than as an intrinsically pro-resolving or M2-directing material. Future studies may expand this approach using primary human macrophages, co-culture systems, and in vivo models to further strengthen the physiological relevance of this experimental platform and enhance its applicability for the evaluation of immunomodulatory biomaterials and their regenerative outcomes. In parallel, the functionalization of PDO-based systems with bioactive factors represents a promising strategy to further modulate immune responses and enhance biological performance. Broader phenotypic and functional profiling across extended time frames will also be important to elucidate how macrophage responses to fibrous PDO evolve and integrate with other cellular processes involved in tissue regeneration.

## Data Availability

The data related to this study are available upon reasonable request to the corresponding author.

## Supplementary Materials

The following supporting information can be downloaded at https://www.mdpi.com/article/10.3390/jfb17090442/s1, Table S1: Statistical analysis results for MTT, CellTiter-Glo^®^, and qRT-PCR datasets.
