# Supplementary material for: Human Macrophage Polarization Dynamics on a 3D Fibrous Polydioxanone Mesh In Vitro
Source: J Funct Biomater. 2026 Sep 2;17(9):442. doi: 10.3390/jfb17090442 (PMC13607951; doi:10.3390/jfb17090442)
Supplement: Supplementary file 1 [file jfb-17-00442-s001.zip › jfb-4317958-supplementary.pdf]

Table S1: Statistical analysis results for MTT, CellTiter-Glo®, and qRT-PCR datasets.

Dataset	Effect	F(df1,df2)	p-value
**MTT**	Polarization condition	F(2,12)=5621.93	<0.0001
	Time	F(1,12)=4373.57	<0.0001
	Interaction	F(2,12)=1376.17	<0.0001
**CellTiter-Glo®**	Polarization condition	F(2,6)=28.62	0.00085
	Time	F(1,6)=1.97	0.2105
	Interaction	F(2,6)=4.81	0.0567
**qRT-PCR IL-8**	Polarization condition	F(2,6)=667.8	<0.0001
	Time	F(1,6)=469.900	<0.0001
	Interaction	F(2,6)=521.40	<0.0001
**qRT-PCR IL-10**	Polarization condition	F(2,6)=14.86	0.0047
	Time	F(1,6)=11.26	0.0153
	Interaction	F(2,6)=11.05	0.0097
**qRT-PCR ARG-2**	Polarization condition	F(2,6)=22.56	0.0016
	Time	F(1,6)=30.21	0.0015
	Interaction	F(2,6)=7.02	0.0269
**qRT-PCR CD206**	Polarization condition	F(2,6)=68.67	<0.0001
	Time	F(1,6)=39.64	0.0007
	Interaction	F(2,6)=75.60	<0.0001

Dataset	Effect	F(df1,df2)	p-value
**qRT-PCR IL-1β**	Polarization condition	F(2,6)=492.90	<0.0001
	Time	F(1,6)=612.60	<0.0001
	Interaction	F(2,6)=476.60	<0.0001
**qRT-PCR IL-18**	Polarization condition	F(2,6)=214.10	<0.0001
	Time	F(1,6)=462.40	<0.0001
	Interaction	F(2,6)=147.5	<0.0001
